# Drivers of Diagnostic Delay in Mitochondrial Disease: Missed Recognition of Canonical Features

**DOI:** 10.1002/jmd2.70068

**Published:** 2026-01-22

**Authors:** Rory J. Tinker, Neil Jacob, Mohammad Ghouse Syed, Janhawi Kelkar, Colleen Donnelly, Ibrahim Elsharkawi, Jaya Ganesh, Bruce D. Gelb, Vikas Pejaver, Tamas Kozicz, Eva Morava

**Affiliations:** ^1^ Department of Medical Genetics and Genomics Icahn School of Medicine at Mount Sinai New York New York USA; ^2^ Institute for Genomic Health, Icahn School of Medicine at Mount Sinai New York New York USA; ^3^ Department of Genetics and Genomic Sciences Icahn School of Medicine at Mount Sinai New York New York USA; ^4^ Mindich Child Health and Development Institute and Departments of Pediatrics and Genetics and Genomic Sciences Icahn School of Medicine at Mount Sinai New York New York USA

**Keywords:** diagnostic delay, electronic health records (EHR), genotype‐first diagnosis, human phenotype ontology (HPO), missed diagnostic opportunities, mitochondrial disease, natural language processing (NLP), phenotype recognition

## Abstract

Diagnostic delay is common in mitochondrial disease, and its drivers remain unclear despite advances in molecular diagnostics. We retrospectively analyzed 61 individuals with molecularly confirmed mitochondrial disease at the Mount Sinai Mitochondrial Disease Clinic, diagnosed after 2016. Diagnostic delay was partitioned into intervals from symptom onset to clinical suspicion, and from suspicion to molecular diagnosis. Demographic, phenotypic, and genetic data were abstracted from health records, and Human Phenotype Ontology terms were compared before and after diagnosis using ClinPhen. Most delays occurred between symptom onset and clinical suspicion (mean 8.17 years) rather than after suspicion (mean 1.28 years), yielding a mean total delay of 8.22 years (median 3.0). Delay decreased sharply by year of birth (*r* = −0.99, *p* < 49.92 × 10^−39^) and symptom onset (*r* = −0.96, *p* < 8.14 × 10^−27^), but showed no meaningful trend with year of diagnosis. Canonical features such as seizures, hypotonia, and stroke were frequently documented years before suspicion, underscoring missed opportunities. Diagnostic delay may reflect missed recognition rather than testing limitations. Systematic capture of early phenotypes and AI/NLP‐based mining of electronic health records could proactively flag patients for reflexive sequencing, shortening diagnostic delay.

## Highlights

- Most of the delay occurred before clinical suspicion: mean onset to clinical suspicion 8.17 years, versus mean suspicion to molecular diagnosis 1.28 years (mean total delay 8.22 years; median 3.0 years).
- Delays have shortened sharply across birth and symptom‐onset cohorts: year of birth versus delay r = −0.99 (*p* < 49.92 × 10^−39^) and symptom‐onset year versus delay r = −0.96 (*p* < 8.14 × 10^−27^), while year of molecular diagnosis showed no association (r = 0.07, *p* = 0.653).
- Canonical features were often documented years before recognition: seizures in 41% with mean lead time 1.8 years before diagnosis; global developmental delay 36% with 3.7 years; hypotonia 36% with 2.1 years; stroke 27% with 4.0 years.

**Abbreviations**
AI: artificial intelligence
EHR: electronic health record
GINA: Genetic Information Nondiscrimination Act
HPO: Human Phenotype Ontology
MELAS: mitochondrial encephalomyopathy, lactic acidosis, and stroke‐like episodes
ML: machine learning
mtDNA: mitochondrial DNA
NLP: natural language processing
WES: whole‐exome sequencing
WGS: whole‐genome sequencing

## 1 Introduction

Primary mitochondrial diseases are a clinically and genetically heterogeneous group of disorders affecting cellular energy metabolism by pathogenic variants in mitochondrial DNA (mtDNA) or nuclear genes [1, 2, 3]. They can involve virtually any organ system, usually with multi‐organ manifestations, although isolated organ involvement can occur. Organ systems most commonly affected include the central nervous system, skeletal muscle, and other high‐energy tissues [4]. Clinical manifestations and age of onset are highly variable, which may delay uniform recognition of these disorders [5]. Diagnostic delays arise because early features are nonspecific, the course of disease is variable, and due to a lack of robust diagnostic biomarkers [6]. Early and accurate diagnosis is essential for patient management, reproductive counseling, and access to emerging therapies, yet mitochondrial disease remains under‐recognized across both pediatric and adult populations [2].

Most existing studies on diagnostic delay in mitochondrial disease originate from single centers or focus on specific syndromic subtypes, often lacking standardized definitions of the diagnostic interval or systematic assessment of associated clinical and demographic factors [7, 8, 9, 10, 11]. A recent scoping review of monogenic disease highlighted substantial variability in study design, outcome measures, and conclusions, revealing that inconsistent definitions of delay severely limit cross‐study comparisons [6]. In parallel, a novel conceptual model applied to Mendelian disease within electronic health records now defines discrete diagnostic intervals (pre‐suspicion, pre‐diagnosis, and post‐diagnosis) as captured by the EHR, enabling systematic study of diagnostic delay in mitochondrial disease [9].

In this pilot study, we examined diagnostic delay in a clinically and genetically diverse cohort of individuals with clinically confirmed mitochondrial disease, using the electronic health record (EHR) to reconstruct the diagnostic trajectory. Our primary outcome was the interval from symptom onset to genetic diagnosis. To better resolve this trajectory, we further partitioned the overall delay into two sequential phases, following a recently proposed conceptual model: (1) the interval from symptom onset to first clinical suspicion of mitochondrial disease and (2) the interval from clinical suspicion to confirmed genetic diagnosis. We examined how these intervals varied by age group, sex, molecular etiology (mtDNA versus nuclear), and clinical phenotype. In addition, we applied natural language processing (NLP) to extract Human Phenotype Ontology (HPO) terms from electronic health records, enabling systematic comparison of phenotype prevalence before and after clinical suspicion for mitochondrial disease. We found that most delays arose before a specific clinical suspicion for a possible mitochondrial diagnosis, that developmental delay was linked to shorter diagnostic intervals, and that canonical features such as seizures, hypotonia, and stroke were often present years before clinical suspicion and diagnosis.

## 2 Methods

### 2.1 Cohort Construction

This study included individuals evaluated at the Mitochondrial Medicine Program at Mount Sinai Hospital. Only patients diagnosed between 2016 and 2025 were included (IRB 25‐00728 Icahn School of Medicine at Mount Sinai Institutional Review Board) [12].

Eligible participants had a confirmed clinical diagnosis of a primary mitochondrial disorder, as determined by a board‐certified clinical biochemical geneticist, requiring concordance between the patient's phenotype and the identified pathogenic mitochondrial DNA, or nuclear DNA variant. All variants were interpreted according to ACMG/AMP guidelines and cross‐referenced with MITOMAP where appropriate.

Heteroplasmy estimation was conducted using mitochondrial DNA sequencing of blood or urine samples, with tissue source determined by clinical sample availability. We excluded individuals with secondary mitochondrial dysfunction due to another primary genetic diagnosis or an acquired condition. Clinical and genetic data were abstracted from the electronic medical record and supplemented by review of gene testing reports. When available, we also collected demographic, clinical, and laboratory variables for exploratory analyses, including sex, genotype category (nuclear vs. mitochondrial), clinical diagnosis, number of similarly affected family members, developmental delay, seizure history, and metabolic abnormalities including elevated lactate. All data were de‐identified before analysis.

### 2.2 Definition of Diagnostic Delay, Clinical Suspicion and Censoring of the Medical Phenome

Diagnostic delay and date of clinical suspension were defined and dated based on a previous model [9]. Diagnostic delay was the time from reported symptom onset to the age at confirmed molecular diagnosis of mitochondrial disease. Symptom onset was abstracted from the medical record using patient or caregiver reports and clinician assessment of when relevant features first appeared. Clinical diagnosis was the date a pathogenic or likely pathogenic variant was identified by genetic testing and confirmed by a clinical genetics provider. Clinical suspicion was the earliest date a clinician documented that a primary mitochondrial disorder or another genetic disorder was being actively considered and warranted evaluation. We identified this date from signed notes or the problem list using explicit phrases such as “suspect mitochondrial disease,” “genetic etiology under consideration,” or a named syndrome, and from plans that initiated targeted evaluation for mitochondrial or genetic disease. When explicit language was absent, we used the earliest strong proxy indicating intent to evaluate for a mitochondrial or genetic disorder, including referral to a mitochondrial clinic or clinical genetics service, ordering mtDNA or nuclear panels or exome or genome sequencing, or starting a diagnostic workup directed at mitochondrial or genetic disease. Dates came from the note timestamp for documentation, the order date for tests, and the referral date for specialty evaluations.

### 2.3 Phenotypic Data Extraction

Clinical notes for each individual were retrieved from the Mount Sinai Health System using the AI‐Ready Mount Sinai (AIR.MS) platform (https://labs.icahn.mssm.edu/airms/). The corpus included progress notes, care notes, telephone encounters, and other document types. Phenotypic features were extracted with ClinPhen, a natural language processing tool that maps clinical text to Human Phenotype Ontology (HPO) terms. HPO term extraction was divided into two windows: features documented before the date of initial clinical suspicion and those recorded after. This separation reduces the inflation of phenotypic features that often occurs once clinicians begin suspecting a diagnosis and helps ensure that extracted HPO profiles reflect true pre‐diagnostic presentation. It also allowed us to directly compare and quantify HPO features occurring before versus after clinical suspicion.

### 2.4 Statistical Analysis

**FIGURE 1:**
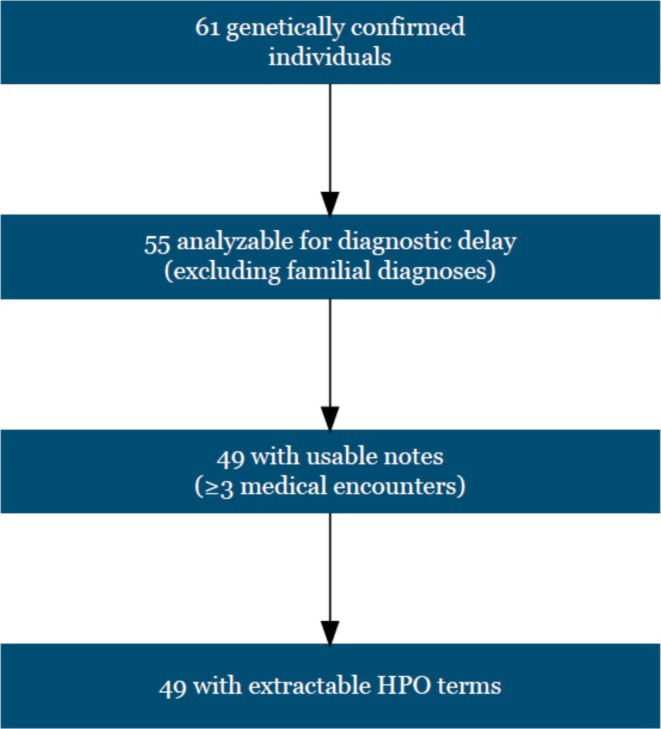
Cohort flow chart and Human Phenotype Ontology (HPO) term extraction. Flow diagram showing cohort selection and phenotypic feature extraction. Of individuals with a clinically confirmed primary mitochondrial disorder, electronic health records were available for 49 patients.

We performed a cross‐sectional analysis of diagnostic delay. Individuals diagnosed through cascade testing were excluded from all delay calculations, as their diagnoses did not reflect an independent clinical evaluation or recognition process. Descriptive statistics were calculated for the overall cohort, including means, standard deviations (SD), standard errors of the mean (SEM), and medians for continuous variables, and frequencies and percentages for categorical variables. To evaluate factors associated with diagnostic delay, variables were categorized into three groups: (1) continuous clinical and demographic variables, (2) binary clinical features (e.g., presence or absence of developmental delay or seizures), and (3) categorical variables with more than two levels (e.g., clinical diagnosis, genetic etiology). Pearson correlation coefficients were used to assess associations between continuous variables and diagnostic delay. Normality of diagnostic delay distributions was assessed using visual inspection and Shapiro–Wilk tests. Diagnostic delay was not normally distributed; therefore, delay was summarized using median and interquartile range. Group comparisons were performed using non‐parametric tests, including the Wilcoxon rank‐sum test for binary variables and the Kruskal–Wallis test for categorical variables with more than two levels. All individuals identified on cascade testing were excluded from delay calculations. Formal multiple‐comparison correction was applied to exploratory analyses presented in the Supporting Information, while primary analyses were interpreted based on effect size and clinical relevance given the limited sample size. To characterize missed diagnostic opportunities, HPO terms were analyzed for frequency and mean lead time (years before suspicion or diagnosis), and their prevalence before and after suspicion was compared. Results were visualized with dumbbell plots and summarized in Supporting Information. All temporal correlations were calculated at the individual level; no averaging by birth‐year strata was performed. Outliers were retained because they represent true variability in diagnostic trajectories, consistent with prior literature [7]. All analyses were conducted in R (version 4.4.1), with results exported into structured Excel files for integration into figures and manuscript tables [13]. For each patient, only the first occurrence of an HPO term was retained for downstream analyses (Figure 1). ClinPhen is openly available (https://github.com/kingmanzhang/clinphen/tree/master) and was run in Python (version 3.11.11). Among the 61 genetically confirmed individuals, clinical note data were available for 49. A total of 20 702 notes were retrieved from 49 individuals, while one individual had no available notes. After restricting to the first occurrence of each term per individual, the dataset comprised 5751 HPO terms across all patients, representing 1489 unique terms.

## 3 Results

### 3.1 Clinical and Genetic Features of the Cohort

This study included 61 individuals with molecularly confirmed mitochondrial disease evaluated through the Mitochondrial Medicine Program at Mount Sinai Hospital. Of these, 55 individuals were eligible for diagnostic‐delay analyses after excluding those diagnosed six diagnosed through familial (cascade) testing. HPO‐based clinical‐note analyses were conducted for 49 individuals with ≥ 3 documented medical encounters. For individuals diagnosed through their symptoms and not families, the age range and mean age at chart review were 0.5–77 years and 22.5 years, respectively. Two individuals were deceased and their ages at death were used. The mean age at the time of genetic diagnosis was 18.83 years (*n* = 55) (Figure 1). The cohort was approximately evenly split by sex, with 29 females and 32 males. Most genetic etiologies in our cohort were mitochondrial DNA–based disorders (62%, *n* = 35), with the remaining cases attributable to nuclear gene–based disorders (38%, *n* = 26). Heteroplasmy among individuals with mitochondrial DNA–based disorders ranged from 5%–100%.

Mitochondrial DNA–based disorders, which accounted for 62% (*n* = 35/61) of cases, were divided into disorders caused by point mutations (97.1%, *n* = 34/35) or by deletions (2.9%, *n* = 1/35). The m.3243A>G variant, classically associated with *mitochondrial encephalomyopathy*, *lactic acidosis*, and *stroke‐like episodes* (MELAS), was the most common (65.7%, *n* = 23/35).

The second most common variant was m.8363G>A (11.7%, *n* = 4/35), typically seen in *myoclonic epilepsy with ragged‐red fibers* (MERRF). Other point mutations included m.3302A>G (MT‐TK, *n* = 1/35), m.9997T>A (MT‐TG, *n* = 1/35), m.3460G>A (MT‐ND1, *n* = 1/35), m.11778G>A (MT‐ND4, *n* = 1/35), m.14484T>C (MT‐ND6, *n* = 1/35), m.3302A>G (tRNA[Leu(UUR)], *n* = 1/35), m.8993T>G (MT‐ATP6, *n* = 1/35), and m.8719G>A (MT‐ATP, *n* = 1/35) (Table 1). Of the individuals with a point mutation, 17.6% (*n* = 6/34) were asymptomatic but were identified with the genetic variant after familial cascade testing. They later developed symptoms. This included four individual families.

**TABLE 1:** Cohort characteristics and clinical subgroups.

Characteristics	Value
Total participants	61
Age range	0.5–77
Mean age (years) (two individuals were deceased, their age at death were used)	21.4
Age at onset, range (years)	0–42
Mean age of onset (years)	10.8
Mean age at genetic diagnosis	18.8
Interval: Onset → clinical suspicion (years)	8.17
Females	29 (47.5%)
Males	32 (52.5%)
Mitochondrial	35 (62%)
Mt. Deletion	1
Mt. Point Mutations	34
m.3243A>G	23
m.8363G>A	4
m.9997 T>A	1
m.11778G>A	1
m.3302A>G	1
m.8993 T>G	1
m.8719G>A	1
m.3460G>A	1
m.14484 T>C	1
Nuclear	26 (38%)
Transport protein	2
SLC25A4	2
Small molecule metabolism	6
ECHS1	3
PC	1
DLD	1
PPA2	1
Complex I	3
NDUFV1	2
NDUFAF1	1
Complex IV	1
SURF1	1
Complex V	1
TMEM70	1
Translation Defects	6
GTPBP3	1
MTO1	1
NARS2	1
TRIT1	1
Mitochondrial Structure/Integrity	3
OPA1	1
TAZ	2
Mitochondrial Maintenance/Integrity	3
POLG	2
TOP3A	1

*Note:* Demographic, genetic, and diagnostic characteristics of the 61 individuals with molecularly confirmed mitochondrial disease evaluated at the Mount Sinai Mitochondrial Disease Clinic. Continuous variables are summarized as mean ± standard deviation (SD). Intervals represent elapsed time between symptom onset, first clinical suspicion, and molecular genetic diagnosis. Sex distribution, genetic etiology (mitochondrial vs. nuclear), and major clinical subgroups are listed with corresponding sample sizes.

Nuclear‐encoded disorders (42.6%, *n* = 26) were subcategorized into defects in protein transport (*SLC25A4*), small molecular metabolism (*ECHS1*, Pyruvate Carboxylase Deficiency, Dihydrolipoamide Dehydrogenase Deficiency, and Inorganic Pyrophosphatase Deficiency), protein translation *(GTPBP3, TRIT1, MTO1, NARS2)*, mitochondrial structure/integrity (*OPA1*, *TAZ*), mitochondrial maintenance/integrity (*POLG, TOP3A*), and nuclear OXPHOS complex function (*NDUFAF1, NDUFV1, SURF1, TMEM70*) (Table 1).

### 3.2 Diagnostic Delay Predominantly Reflects Lag in Clinical Suspicion

**FIGURE 2:**
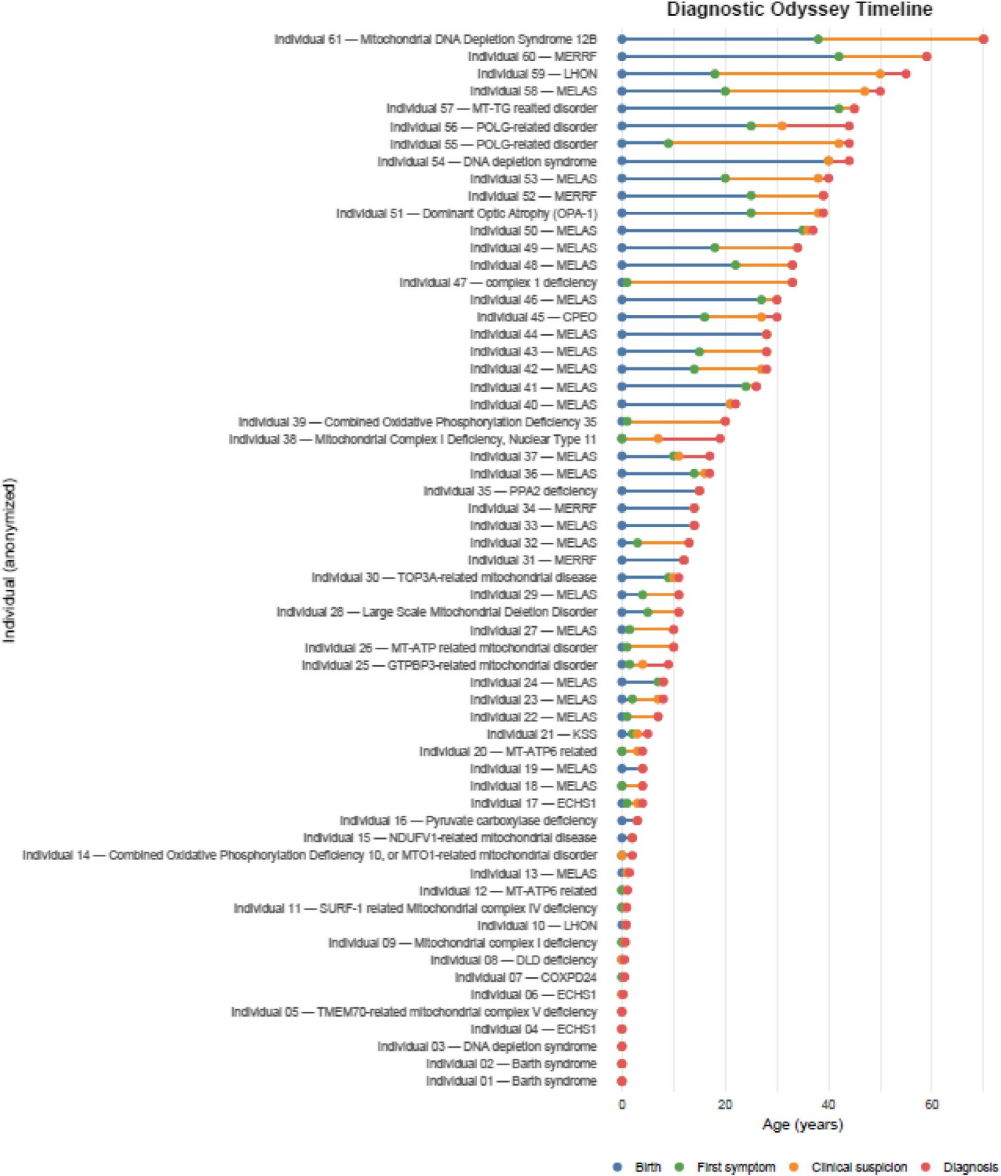
Diagnostic odyssey timeline of individuals with mitochondrial disease. Each horizontal bar represents a single participant, grouped by clinical diagnosis, showing the timeline from birth to key diagnostic milestones. The figure highlights the substantial variability in diagnostic trajectories across individuals, with most of the delay arising during the interval from symptom onset to clinical suspicion.

The mean delay was 8.17 years (SD: 9.9, SEM: 1.29; *n* = 55), highlighting the prolonged but variable time to diagnosis experienced by many individuals with mitochondrial disease (Figure 2). The mean age of symptom onset was 10.8 years (*n* = 55) with clinical suspicion typically raised at a mean age of 17.7 years (*n* = 55). On average, the interval from first symptom onset to clinical suspicion was a delay of 7.36 years, while the interval from clinical suspicion to molecular diagnosis was 1.28 years. These diagnostic timing data are summarized in Table 1 and graphically represented in Figure 2.

### 3.3 Birth and Onset Year Predict Shorter Delays, While Diagnosis Year Does Not

**FIGURE 3:**
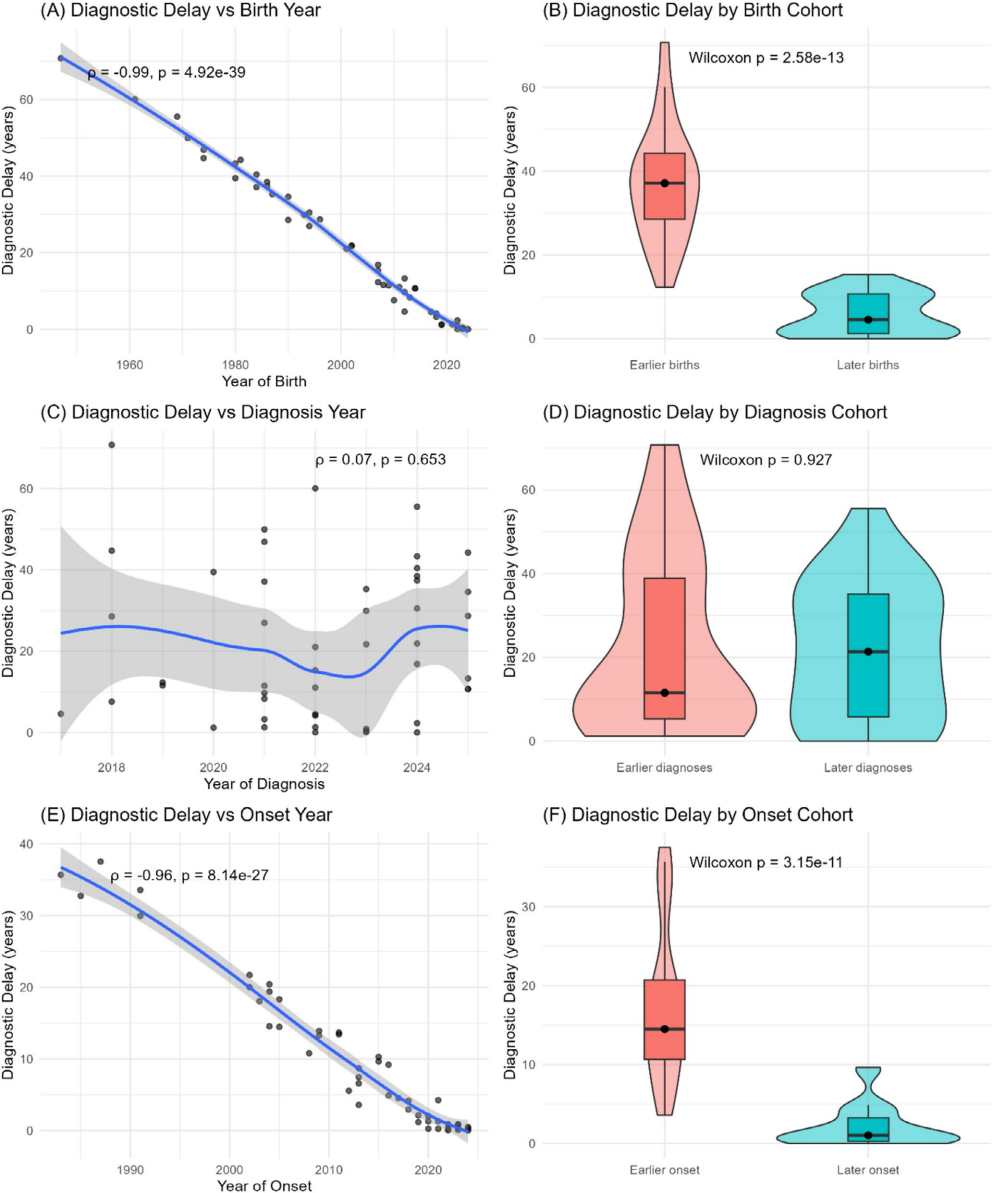
Trends in diagnostic delay across birth year, diagnosis year, and symptom onset. (A) Diagnostic delay was strongly negatively correlated with year of birth, with later birth cohorts experiencing shorter delays. (B) Patients born in the latter half of the cohort had significantly shorter delays compared with those born earlier. (C) Diagnostic delay showed no significant association with year of molecular diagnosis. (D) Splitting by earlier versus later diagnosis year revealed no difference in delay. (E) Diagnostic delay was strongly negatively correlated with year of symptom onset. (F) Patients with later symptom onset experienced shorter delays compared with those with earlier onset. Together, these panels show that diagnostic delays have decreased substantially over time, primarily due to differences in birth cohort and age of symptom onset, rather than year of diagnosis.

Diagnostic delay has shortened substantially across successive generations of patients with mitochondrial disease. As shown in Figure 3A, there was a strong inverse correlation between year of birth and diagnostic delay (*r* = −0.99, *p* < 49.92 × 10^–39^), with later‐born individuals experiencing markedly shorter delays. This pattern was confirmed when the cohort was stratified into earlier versus later groups using the median birth year, median year of symptom onset, and median year of diagnosis for interpretability (birth year median = 2007; onset year median = 2015 diagnosis year median = 2022). Individuals falling before each respective median were classified as “early,” and those after as “late.” Across all three‐time anchors, earlier cohorts consistently experienced substantially longer diagnostic delays compared with later cohorts (Figure 3B, *p* = 2.58 × 10^−13^).

In contrast, the year of molecular diagnosis was not associated with diagnostic delay (Figure 3C, *r* = 0.07, *p* = 0.653), and splitting the cohort by earlier versus later diagnosis years did not reveal a difference (Figure 3D, *p* = 0.93). Instead, the key driver of improvement was the era of symptom onset: diagnostic delay was strongly negatively correlated with year of onset (Figure 3E, *r* = −0.96, *p* < 8.14 × 10^−27^), and individuals with later‐onset symptoms had significantly shorter delays compared to those with earlier onset (Figure 3F, *p* = 3.15 × 10^−11^). Collectively, these findings indicate that diagnostic delays have decreased over time, largely due to changes in when symptoms arise and are first recognized, rather than improvements tied directly to the calendar year of diagnosis.

### 3.4 Factors Positively Associated With Increased Diagnostic Delay

**FIGURE 4:**
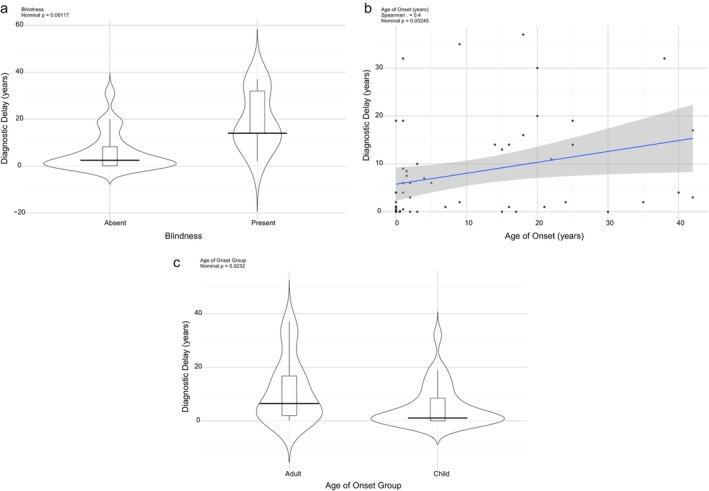
Significant clinical and demographic features associated with diagnostic delay. Plots showing features significantly associated with diagnostic delay in mitochondrial disease. Brainstem involvement and developmental delay were associated with shorter diagnostic delays, while visual loss was associated with longer delays. Younger age of onset correlated with shorter delay, whereas older onset correlated with longer delay. Scatter, violin, and box plots illustrate distributions with mean and median estimates, and *p*‐values are from *t*‐tests or Pearson correlation as appropriate.

We next examined demographic and clinical factors that might predict variation in diagnostic delay (Figure 4). Several features emerged as significant predictors. Visual loss was associated with significantly longer delays (Median 14 affected vs. 2.5 unaffected, *p* = 0.001). Later age of symptom onset correlated with longer diagnostic delays (*r* = 0.4, *p* = 0.002), suggesting that mitochondrial disease presenting in older individuals is more easily overlooked. By contrast, neither sex nor the broad category of nuclear versus mitochondrial DNA genetic etiology was associated with time to diagnosis. Several additional variables were tested but were not significant predictors, including common clinical features (such as seizures, cardiomyopathy, and muscle weakness), laboratory parameters, and age at first presentation. These non‐significant associations are shown in Table S1.

### 3.5 Sub‐Analysis of Familial Cases

Of the 61 cases, six were identified through familial cascade testing (Table S2). The mean age at diagnosis in these cases was 14.4 years, earlier than in non‐familial cases though not statistically significant (18.5 years; *p* < 0.40, unpaired *t*‐test). The chart review showed that although not clinically diagnosed at the time of diagnosis, all individuals went on to have symptoms.

### 3.6 Documented HPO Features Reveal Missed Diagnostic Opportunities

**FIGURE 5:**
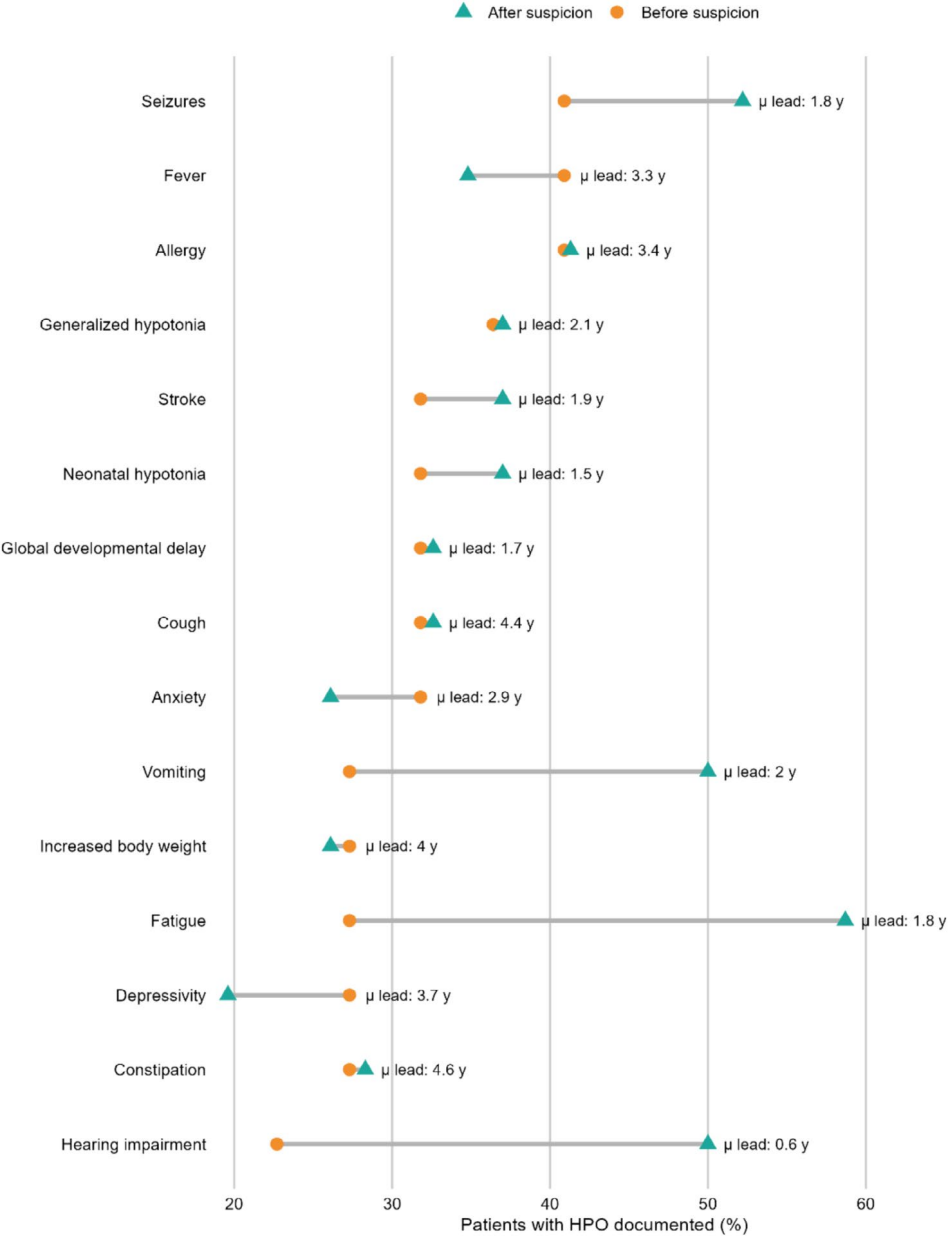
HPO features documented before versus after clinical suspicion of mitochondrial disease. Dumbbell plot showing the proportion of patients with the most common selected Human Phenotype Ontology (HPO) terms documented before (circle, left) and after (triangle, right) the point of clinical suspicion for mitochondrial disease. Features such as seizures, global developmental delay, hypotonia, and stroke were frequently recorded years in advance, while nonspecific features (e.g., fatigue, constipation, anxiety) were also common but less clearly interpreted as disease‐related. Horizontal connectors illustrate persistence of symptoms across both periods, highlighting missed opportunities for earlier recognition. Mean lead time (years prior to diagnosis) is displayed adjacent to each feature.

To quantify differential documentation before versus after clinical suspicion, we compared HPO term and clinical note counts across these intervals. Of 4400 extracted HPO terms, 19.7% occurred before suspicion and 80.3% after. Similarly, of clinical notes, 19.6% were written before suspicion and 80.4% after. At the patient level, the median number of notes increased from three before suspicion to 15.5 after, and HPO density increased from a median of 22 to 47 terms. Analysis of clinical notes revealed that several key HPO terms were frequently present in the medical record well before mitochondrial disease was suspected and diagnosed (Figure 5, Table S3). Canonical features such as seizures (mean 1.8 years before diagnosis; 41% of individuals), global developmental delay (3.7 years; 36%), hypotonia (2.1 years; 36%), and stroke (4.0 years; 27%) were consistently documented years in advance, highlighting a substantial diagnostic window. Other features, including neonatal hypotonia (4.6 years; 23%), hearing impairment (0.6 years; 18%), vomiting (1.9 years; 23%), and increased body weight (1.7 years; 27%), were also recorded prior to suspicion and often persisted. By contrast, nonspecific symptoms such as fatigue, constipation, cough, anxiety, and depression were common but less clearly disease‐related, with shorter lead times and limited specificity. Together, these findings demonstrate that diagnostically informative phenotypes were often present in the EHR long before recognition, suggesting that systematic capture and flagging of such terms could substantially shorten diagnostic delays.

## 4 Discussion

In this study of 58 individuals molecularly diagnosed with mitochondrial disease evaluated at a tertiary referral center between 2016–2025, we found that diagnostic delay remains substantial, with a mean of 8.22 years from symptom onset to diagnosis. Most of this delay (90.1% of the time) occurred before clinical suspicion was raised, while the time from suspicion to diagnosis was comparatively short. Delays have shortened across generations, with later‐born individuals and those with more recent symptom onset experiencing significantly faster diagnoses. These temporal patterns likely reflect changes in the genetic testing landscape, with the rise in diagnoses after 2010 coinciding with the adoption of next‐generation sequencing, expanded mitochondrial gene panels, and improved clinical recognition. Accordingly, individuals with more recent birth or onset years tended to experience shorter diagnostic delays, suggesting that observed correlations may reflect evolving diagnostic pathways rather than biologically implausible effects. Phenotype‐specific predictors also emerged: brainstem involvement and developmental delay were associated with shorter delays, whereas visual loss and later age of symptom onset were associated with longer delays. It's unclear from the current study if this shortening is due to improved sequencing or improved clinical recognition, although we speculate both factors are contributory. Finally, analysis of Human Phenotype Ontology (HPO) terms demonstrated that diagnostically informative features, including seizures, hypotonia, developmental delay, and stroke were frequently documented years before suspicion, underscoring a wide diagnostic window and repeated missed opportunities for recognition [14, 15, 16]. Despite the expected post‐suspicion inflation in documentation volume and phenotypic richness, key mitochondrial features were already present in many patients before clinical suspicion, indicating that delays stemmed from missed recognition rather than absence of early phenotypes.

Diagnostic odysseys contribute to patient morbidity, and in some cases mortality, as well as patient frustration, mental health exacerbations, and a rise in healthcare costs. In this study, five individuals from four families were diagnosed with primary mitochondrial disease through cascade testing after symptom onset in a close relative, highlighting both the utility and the ethical complexity of testing unaffected family members [17]. This may be further compounded by variable penetrance and variable expressivity seen across many different primary mitochondrial diseases [18]. Heteroplasmy levels varied widely between asymptomatic (46%–92% in blood; 55% in urine) and affected relatives (39%–95%), underscoring the unpredictable relationship between genotype and phenotype. For example, a 7‐year‐old who suffered a cerebral infarction during an orthopedic procedure was found to carry the m.3243A>G MELAS variant at 77% heteroplasmy in blood, prompting consideration of testing his asymptomatic siblings, both under age 15. While prophylactic arginine may have a limited role in modulating stroke risk, evidence for its use and efficacy is lacking; this raises questions about the clinical utility of testing asymptomatic minors, particularly when heteroplasmy complicates prognostication and a positive result may cause anxiety, stigma, or fear of discrimination despite protections such as the Genetic Information Nondiscrimination Act [19, 20]. The five cascade cases likely underestimate the true prevalence, as some relatives decline testing [21]. More broadly, diagnostic timelines have improved across generations, likely driven more by the widespread adoption of exome and genome sequencing than by earlier clinical recognition [22]. By applying a conceptual model that partitions the diagnostic odyssey into pre‐ and post‐suspicion intervals, we showed that most delay occurs before suspicion, when canonical features are present but unrecognized. Leveraging EHR‐derived HPO terms with natural language processing further demonstrated how diagnostically informative phenotypes are systematically overlooked in real time. Taken together, these findings echo prior literature on the variability and inconsistency of diagnostic trajectories in monogenic disease, while also pointing to priority areas for intervention, ranging from ethical frameworks for cascade testing to the development of AI‐driven tools that could assist recognition when suspicion is absent [23, 24, 25].

This study has several limitations. First, it reflects a single‐center cohort from a tertiary referral clinic, which may bias toward more complex or severe cases and limit generalizability. Access to testing and awareness may differ across health systems, underscoring questions about equity and healthcare access across different socioeconomic, geographical, and demographic groups [26]. Second, dates of symptom onset and suspicion were abstracted from medical record to inconsistent documentation, and fragmentation across health systems [27]. Although we used a standardized definition of clinical suspicion, its ascertainment inevitably relied on documentation practices that may vary between clinicians and specialties. Third, while we applied NLP for systematic HPO extraction, missingness or data fragmentation in clinical notes and variation in terminology could underestimate the true prevalence or lead time of some features. Furthermore, a detailed assessment of referral pathways was not possible because internal versus external referral information was inconsistently documented. Similarly, insurance authorization and access‐related barriers could not be reliably evaluated. In addition, the cohort includes a high proportion of individuals with the m.3243A>G variant, which may limit the generalizability of some symptom‐based findings given the characteristic multisystem phenotype associated with this genotype. Additionally, the modest sample size—an inherent limitation of clinically ascertained rare‐disease cohorts—restricts genotype‐specific stratification, use of complex statistical models, and increases the risk of type I error, requiring interpretation in the context of Bonferroni‐corrected results (Table S1).

Despite these limitations, this study highlights several key implications. First, improving recognition of mitochondrial disease requires tools and training to ensure that canonical features documented in the EHR, such as seizures, hypotonia, and stroke, trigger earlier suspicion. Second, our findings provide retrospective evidence for the potential of a genotype‐first approach, in which sequencing is pursued reflexively when certain combinations of phenotypes are present, thereby bypassing delays in clinical recognition. This approach may be complicated by the presence of variants of unknown significance, the incomplete ability of genetic sequencing alone to catch all primary mitochondrial diseases, and the challenges with mtDNA variant and heteroplasmy interpretation. Third, the successful use of NLP‐based Human Phenotype Ontology extraction illustrates how automated phenotypic modeling could be deployed in real time to flag patterns highly suggestive of mitochondrial disease, prompting earlier referral and testing [23, 28, 29]. Fourth, the marked reduction in diagnostic delays across generations suggests that earlier recognition is possible and may be further accelerated through awareness campaigns, integration of phenotype‐driven decision support, and expanded access to sequencing. Fifth, by constructing a large, systematically annotated but censored dataset, this study provides a foundation for the development and validation of machine learning and AI tools aimed at shortening diagnostic delay in the future. Future directions of this work may involve integrating phenotype recognition algorithms in EHR as well as targeted educational outreach across different specialties. Finally, these findings align with broader calls to standardize definitions of diagnostic delay and to apply conceptual frameworks across rare genetic diseases, thereby enabling benchmarking, cross‐cohort comparisons, and evaluation of interventions designed to shorten the diagnostic odyssey.

## 5 Conclusion

In summary, diagnostic delays in mitochondrial disease appear to stem largely from missed recognition of canonical features, although the evidence cannot fully disentangle contributions from pediatric symptom profiles, evolving diagnostic pathways across birth cohorts, increased availability of sequencing, and changing referral patterns. By applying a standardized framework and leveraging EHR‐derived phenotypes, this study highlights how genotype‐first approaches and automated phenotypic modeling may help shorten the diagnostic odyssey. Integrating such tools into clinical practice has the potential to support earlier, more equitable diagnosis and improved care for individuals with mitochondrial disease.

## Funding

The authors have nothing to report.

## Conflicts of Interest

The authors declare no conflicts of interest.

## Supporting information


**Table S1:** Predictors of diagnostic delay in mitochondrial disease. Summary of demographic, genetic, and clinical features tested for association with diagnostic delay. The table reports sample size, test statistics, and *p*‐values for each factor.

**Table S2:** Family demographics of individuals who were asymptomatic but were tested because of family history. The proband was the individual who presented with symptoms and initiated cascade testing. The mother of Family 3 had an affected brother who was not seen in our clinic. DM2, Diabetes Mellitus Type II; GDD, Global Developmental Delay; N/A, Asymptomatic Individuals; SNHL, Sensorineural Hearing Loss.

**Table S3:** Frequency and lead time of HPO terms documented prior to clinical suspicion Summary of all HPO terms identified in the electronic health record (EHR) with documentation before and after the point of clinical suspicion for mitochondrial disease. For each feature, the table provides the proportion of patients with documentation before and after suspicion, the number of patients contributing lead time data, and the mean and median years the feature was recorded prior to diagnosis. Inclusion in the analysis required that each individual have at least one HPO term documented before suspicion, to account for data fragmentation across records.

## Data Availability

The data that support the findings of this study are available on request from the corresponding author. The data are not publicly available due to privacy or ethical restrictions.

## References

[1] H. Geiger , Y. Furuta , S. van Wyk , J. A. Phillips , and R. J. Tinker , “The Clinical Spectrum of Mosaic Genetic Disease,” Genes 15 (2024): 1240, 10.3390/genes15101240. 39457364 PMC11507335

[2] R. J. Tinker , A. Z. Lim , R. J. Stefanetti , and R. McFarland , “Current and Emerging Clinical Treatment in Mitochondrial Disease,” Molecular Diagnosis & Therapy 25 (2021): 181–206, 10.1007/s40291-020-00510-6. 33646563 PMC7919238

[3] G. S. Gorman , P. F. Chinnery , S. DiMauro , et al., “Mitochondrial Diseases,” Nature Reviews. Disease Primers 2 (2016): 16080, 10.1038/nrdp.2016.80. 27775730

[4] H. Wen , H. Deng , B. Li , et al., “Mitochondrial Diseases: From Molecular Mechanisms to Therapeutic Advances,” Signal Transduction and Targeted Therapy 10 (2025): 9, 10.1038/s41392-024-02044-3. 39788934 PMC11724432

[5] R. J. Tinker , M. J. Falk , A. Goldstein , et al., “Early Developmental Delay in Leigh Syndrome Spectrum Disorders Is Associated With Poor Clinical Prognosis,” Molecular Genetics and Metabolism 135 (2022): 342–349, 10.1016/j.ymgme.2022.02.006. 35216885 PMC8965798

[6] R. J. Tinker , M. Fisher , A. F. Gimeno , et al., “Diagnostic Delay in Monogenic Disease: A Scoping Review,” Genetics in Medicine 26 (2024): 101074, 10.1016/j.gim.2024.101074. 38243783 PMC11140588

[7] P. Forny , E. Footitt , J. E. Davison , et al., “Diagnosing Mitochondrial Disorders Remains Challenging in the Omics Era,” Neurology Genetics 7 (2021): e597, 10.1212/NXG.0000000000000597. 34056100 PMC8161540

[8] X. Zhao , M. Yu , W. Zhang , Y. Hou , Y. Yuan , and Z. Wang , “Demographic Characteristics, Diagnostic Challenges, Treatment Patterns, and Caregiver Burden of Mitochondrial Diseases: A Retrospective Cross‐Sectional Study,” Orphanet Journal of Rare Diseases 19 (2024): 287, 10.1186/s13023-024-03289-5. 39095827 PMC11297657

[9] R. J. Tinker , J. Peterson , and L. Bastarache , “Phenotypic Presentation of Mendelian Disease Across the Diagnostic Trajectory in Electronic Health Records,” Genetics in Medicine 25 (2023): 100921, 10.1016/j.gim.2023.100921. 37337966 PMC11092403

[10] J. L. P. Thompson , A. Karaa , H. Pham , et al., “The Evolution of the Mitochondrial Disease Diagnostic Odyssey,” Orphanet Journal of Rare Diseases 18 (2023): 157, 10.1186/s13023-023-02754-x. 37349818 PMC10288668

[11] J. Grier , M. Hirano , A. Karaa , E. Shepard , and J. L. P. Thompson , “Diagnostic Odyssey of Patients With Mitochondrial Disease,” Neurology Genetics 4 (2018): e230, 10.1212/NXG.0000000000000230. 29600276 PMC5873725

[12] Mitochondrial Medicine Program , Mount Sinai Health System, https://www.mountsinai.org/care/genetics/services/mitochondrial‐medicine.

[13] C. A. Deisseroth , J. Birgmeier , E. E. Bodle , et al., “ClinPhen Extracts and Prioritizes Patient Phenotypes Directly From Medical Records to Expedite Genetic Disease Diagnosis,” Genetics in Medicine 21 (2019): 1585–1593, 10.1038/s41436-018-0381-1. 30514889 PMC6551315

[14] K. Singh , I. N. Singh , E. Diggins , et al., “Developmental Regression and Mitochondrial Function in Children With Autism,” Annals of Clinical Translational Neurology 7 (2020): 683–694, 10.1002/acn3.51034. 32343046 PMC7261756

[15] L. P. Braz , Y. S. Ng , G. S. Gorman , et al., “Neuromuscular Junction Abnormalities in Mitochondrial Disease,” Neurology Clinical Practice 11 (2021): 97–104, 10.1212/CPJ.0000000000000795. 33842062 PMC8032443

[16] C. Pizzamiglio , E. Bugiardini , W. L. Macken , C. E. Woodward , M. G. Hanna , and R. D. S. Pitceathly , “Mitochondrial Strokes: Diagnostic Challenges and Chameleons,” Genes (Basel) 12 (2021): 1643, 10.3390/genes12101643. 34681037 PMC8535945

[17] S. Haque , K. Crawley , D. Schofield , R. Shrestha , and C. M. Sue , “Cascade Testing in Mitochondrial Diseases: A Cross‐Sectional Retrospective Study,” BMC Neurology 24 (2024): 343, 10.1186/s12883-024-03850-6. 39272026 PMC11396135

[18] R. J. Tinker , L. Bastarache , K. Ezell , et al., “The Contribution of Mosaicism to Genetic Diseases and de Novo Pathogenic Variants,” American Journal of Medical Genetics. Part A 191 (2023): 2482–2492, 10.1002/ajmg.a.63309. 37246601 PMC11167532

[19] R. D. Ganetzky and M. J. Falk , “8‐Year Retrospective Analysis of Intravenous Arginine Therapy for Acute Metabolic Strokes in Pediatric Mitochondrial Disease,” Molecular Genetics and Metabolism 123 (2018): 301–308, 10.1016/j.ymgme.2018.01.010. 29428506 PMC5849405

[20] C. R. Chapman , K. S. Mehta , B. Parent , and A. L. Caplan , “Genetic Discrimination: Emerging Ethical Challenges in the Context of Advancing Technology,” Journal of Law and the Biosciences 7 (2019): lsz016, 10.1093/jlb/lsz016. 34221431 PMC8249090

[21] R. M. Kahn , M. D. Ahsan , E. Chapman‐Davis , et al., “Barriers to Completion of Cascade Genetic Testing: How Can We Improve the Uptake of Testing for Hereditary Breast and Ovarian Cancer Syndrome?,” Familial Cancer 22 (2023): 127–133, 10.1007/s10689-022-00316-x. 36207653 PMC10947313

[22] L. Bastarache , R. J. Tinker , B. A. Schuler , et al., “Characterizing Trends in Clinical Genetic Testing: A Single‐Center Analysis of EHR Data From 1.8 Million Patients Over Two Decades,” American Journal of Human Genetics 112 (2025): 1029–1038, 10.1016/j.ajhg.2025.03.009. 40245861 PMC12120179

[23] C. Shyr , T. A. Cassini , R. J. Tinker , et al., “Large Language Models for Rare Disease Diagnosis at the Undiagnosed Diseases Network,” JAMA Network Open 8 (2025): e2528538, 10.1001/jamanetworkopen.2025.28538. 40844783 PMC12374213

[24] T. Groza , C.‐H. Chan , D. A. Pearce , and G. Baynam , “Realising the Potential Impact of Artificial Intelligence for Rare Diseases: A Framework,” Rare 3 (2025): 100057, 10.1016/j.rare.2024.100057.

[25] C. M. Wilczewski , J. Obasohan , J. E. Paschall , et al., “Genotype First: Clinical Genomics Research Through a Reverse Phenotyping Approach,” American Journal of Human Genetics 110 (2023): 3–12, 10.1016/j.ajhg.2022.12.004. 36608682 PMC9892776

[26] N. A. Borja , R. J. Tinker , S. A. Bivona , et al., “Advancing Equity in Rare Disease Research: Insights From the Undiagnosed Disease Network,” American Journal of Medical Genetics. Part A 197 (2025): e63904, 10.1002/ajmg.a.63904. 39400494 PMC11698638

[27] W.‐Q. Wei , C. L. Leibson , J. E. Ransom , et al., “Impact of Data Fragmentation Across Healthcare Centers on the Accuracy of a High‐Throughput Clinical Phenotyping Algorithm for Specifying Subjects With Type 2 Diabetes Mellitus,” Journal of the American Medical Informatics Association 19 (2012): 219–224, 10.1136/amiajnl-2011-000597. 22249968 PMC3277630

[28] M. M. Shuey , W. W. Stead , I. Aka , et al., “Next‐Generation Phenotyping: Introducing phecodeX for Enhanced Discovery Research in Medical Phenomics,” Bioinformatics 39 (2023): btad655, 10.1093/bioinformatics/btad655. 37930895 PMC10627409

[29] C. Shyr , R. J. Tinker , P. A. Harris , et al., “Accuracy of Large Language Models in Generating Rare Disease Differential Diagnosis Using Key Clinical Features,” Studies in Health Technology and Informatics 329 (2025): 1054–1058, 10.3233/SHTI251000. 40776018 PMC13075642

