# Supplementary material for: Drivers of Diagnostic Delay in Mitochondrial Disease: Missed Recognition of Canonical Features
Source: JIMD Rep. 2026 Jan 22;67(1):e70068. doi: 10.1002/jmd2.70068 (PMC12827488; doi:10.1002/jmd2.70068)
Supplement: Supplementary file 2 — Table S2: Family demographics of individuals who were asymptomatic but were tested because of family history. The proband was the individual who presented with symptoms and initiated cascade testing. The mother of Family 3 had an affected brother who was not seen in our clinic. DM2, Diabetes Mellitus Type II; GDD, Global Developmental Delay; N/A, Asymptomatic Individuals; SNHL, Sensorineural Hearing Loss. [file JMD2-67-e70068-s002.docx]

Supplement Table 2

| **Familial Cases:** | Heteroplasmy (blood) | Presenting Symptom |
| --- | --- | --- |
| m.3243A>G Family 1: n=3 |  |  |
| Mother | 21% | DM2 |
| Daughter | 55% (urine) | N/A |
| **Son (Proband)** | **39%** | **Migraines** |
| m.3243A>G Family 2: n=3 |  |  |
| **Proband** | **77%** | **Stroke** |
| Sister | 46% | N/A |
| Brother | 47% | N/A |
| m.3243A>G Family 3: n=3 |  |  |
| Mother | 34% | SNHL |
| Daughter 1 | 60% | N/A |
| Daughter 2 | 77% | GDD, hypotonia, exercise intolerance |
| m.8363G>A Family 4: n=3 |  |  |
| **Mother (Proband)** | 95% | Ataxia, SNHL |
| Daughter 1 | 92% | N/A |
| Daughter 2 | 86% | N/A |
| m.14484T>C (Incidental Finding) | 100% | N/A (Tested for Congenital Arthrogryposis) |
